# The Science behind the Probiotic Strain *Bifidobacterium animalis* subsp. *lactis* BB-12^®^

**DOI:** 10.3390/microorganisms2020092

**Published:** 2014-03-28

**Authors:** Mikkel Jungersen, Anette Wind, Eric Johansen, Jeffrey E. Christensen, Birgitte Stuer-Lauridsen, Dorte Eskesen

**Affiliations:** 1 Scientific Affairs, Chr Hansen A/S, Hørsholm DK-2970, Denmark; E-Mail: dkdoe@chr-hansen.com; 2 Cultures and Enzymes Division-Innovation, Chr Hansen A/S, Hørsholm DK-2970, Denmark; E-Mails: dkawi@chr-hansen.com (A.W.); dkejo@chr-hansen.com (E.J.); dkbsl@chr-hansen.com (B.S.-L.); 3 Health and Nutrition Division-Innovation, Chr Hansen A/S, Hørsholm DK-2970, Denmark; E-Mail: dkjyc@chr-hansen.com

**Keywords:** probiotic, *Bifidobacterium*, BB-12^®^

## Abstract

This review presents selected data on the probiotic strain *Bifidobacterium animalis* subsp. *lactis* BB-12^®^ (BB-12^®^), which is the world’s most documented probiotic *Bifidobacterium*. It is described in more than 300 scientific publications out of which more than 130 are publications of human clinical studies. The complete genome sequence of BB-12^®^ has been determined and published. BB-12^®^ originates from Chr. Hansen’s collection of dairy cultures and has high stability in foods and as freeze dried powders. Strain characteristics and mechanisms of BB-12^®^ have been established through extensive *in vitro* testing. BB-12^®^ exhibits excellent gastric acid and bile tolerance; it contains bile salt hydrolase, and has strong mucus adherence properties, all valuable probiotic characteristics. Pathogen inhibition, barrier function enhancement, and immune interactions are mechanisms that all have been demonstrated for BB-12^®^. BB-12^®^ has proven its beneficial health effect in numerous clinical studies within gastrointestinal health and immune function. Clinical studies have demonstrated survival of BB-12^®^ through the gastrointestinal tract and BB-12^®^ has been shown to support a healthy gastrointestinal microbiota. Furthermore, BB-12^®^ has been shown to improve bowel function, to have a protective effect against diarrhea, and to reduce side effects of antibiotic treatment, such as antibiotic-associated diarrhea. In terms of immune function, clinical studies have shown that BB-12^®^ increases the body’s resistance to common respiratory infections as well as reduces the incidence of acute respiratory tract infections.

## 1. Introduction

### 1.1. The Microbiota

The human body consists of approximately 10 trillion cells, each encoding approximately 23,000 genes. We are, however, outnumbered by our microbiome—the bacteria living on and in us. The microbiome is made up of more than 500 different species and accounts for around 100 trillion cells encoding 3.3 million different genes. Not surprisingly, the microbiome plays a major role in human health through intimate interaction with the body. The bacteria living in the intestine—the gastrointestinal microbiota—constitute the largest part of the microbiome.

Research on the gastrointestinal microbiota, as well as of probiotics, has increased significantly in the new millennium and the interaction between the gastrointestinal microbiota and probiotics—beneficial bacteria—has gained considerable awareness. Clinical research has shown that probiotics play a role within various health areas, of which the two main research areas are gastrointestinal health and immune function.

### 1.2. Probiotics

The word probiotic is derived from Greek and means “for life”, as opposed to antibiotics which means “against life”. Probiotics are defined as “*live microorganisms, which when administered in adequate amounts, confer a health benefit on the host*” [1]. From this definition it is evident that a probiotic requires that some prerequisites be fulfilled.

First, probiotics need to be alive at the time of ingestion and they must be microorganisms. At present, most known probiotic organisms are bacteria, belonging to the *Lactobacillus* and *Bifidobacterium* genera.

Second, they need to be ingested in a dosage high enough to cause an effect. The recommended, efficacious dosage is closely linked to the clinical documentation, on which it must be based.

Third, the ingested live microorganisms need to confer a beneficial effect on the host. It is important to note that the beneficial effects of a given probiotic is specific to that strain, and cannot be regarded as general for other strains of the same species, or other species, of bacteria or yeast.

### 1.3. Strain Level

The consensus of strain specificity is based on research showing that various strains within the same species may display different effects. In order to establish a probiotic strain it is therefore essential to document characteristics, safety, and efficacy of the specific probiotic strain.

## 2. Taxonomy & Characterization

### 2.1. Taxonomy

*Bifidobacterium* is a genus of lactic acid producing, Gram-positive, non-spore forming, non-motile, anaerobic bacteria. Bifidobacteria were first discovered and isolated from the feces of a breast fed infant in 1899. They are common constituents of the indigenous microbiota in the human intestinal tract [2].

*Bifidobacterium* BB-12^®^ (BB-12^®^) is a catalase-negative, rod-shaped bacterium. It was deposited in the cell culture bank of Chr. Hansen in 1983. At the time of isolation, BB-12^®^ was considered to belong to the species *Bifidobacterium bifidum*. Modern molecular classification techniques reclassified BB-12^®^ as *Bifidobacterium animalis* and later to a new species *Bifidobacterium lactis*. The species *B. lactis* was later shown not to fulfill the criteria for a species and was instead included in *Bifidobacterium animalis* as a subspecies. Today, BB-12^®^ is therefore classified as *Bifidobacterium animalis* subsp. *lactis*. Despite a change in the name over the years, the strain BB-12^®^ has not changed.

### 2.2. Origin & Selection

BB-12^®^ originates from Chr. Hansen’s collection of dairy cultures. It is a strain that was specially selected by Chr. Hansen for the production of probiotic dairy products. BB-12^®^ has been used in infant formula, dietary supplements and fermented milk products worldwide. This strain is technologically well suited, expressing fermentation activity, high aerotolerance, good stability and a high acid and bile tolerance, also as freeze-dried products in dietary supplements. Furthermore, BB-12^®^ does not have adverse effects on taste, appearance or on the mouth feel of the food and is able to survive in the probiotic food until consumption.

### 2.3. The Genome

The properties of an organism are encoded in its DNA with the full complement of DNA in an organism being referred to as its genome. DNA sequencing technology has advanced to the point where it is possible to determine the complete genome sequence of any organism. The information hidden in the genome of a bacterial strain is fundamental for full characterization of the strain and for thorough explorations of its mechanisms and potential as a probiotic.

In 2002, a research project was initiated to determine the genome sequence of BB-12^®^ and by 2004 the complete genome of BB-12^®^ was mapped. This project was presented at a scientific congress and published in 2005 [3] and the complete genome sequence was published in 2010 [4].

**Figure 1:**
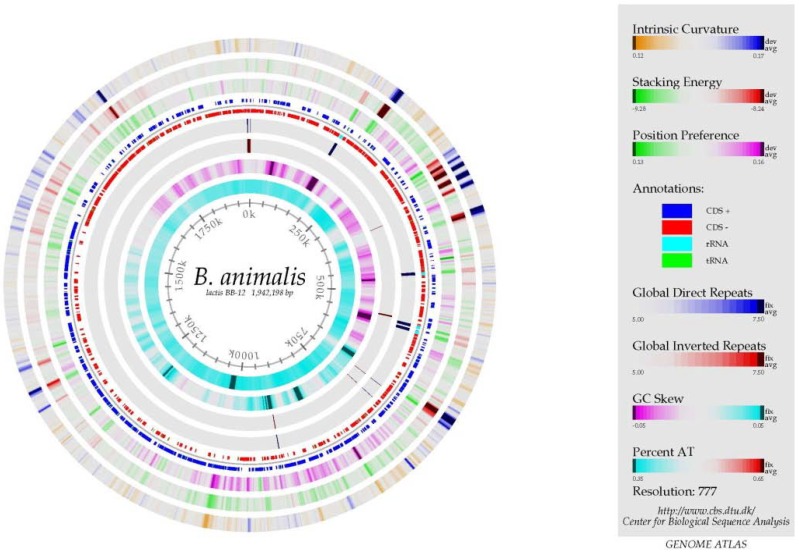
The BB-12^®^ genome atlas. The physical mapping of the BB-12^®^ chromosome revealed that the genome sequence was correctly assembled. Reproduced from Garrigues *et al*. 2013 [6] with permissions from Elsevier, Copyright 2013.

The BB-12^®^ genome consists of a single circular chromosome of 1,942,198 base pairs with 1642 predicted protein-encoding genes, 4 rRNA operons, and 52 tRNA genes. A physical mapping of the BB-12^®^ chromosome revealed that the genome sequence was correctly assembled (Figure 1). One important use of genome sequence information is that it allows a comparison to the genomes of other organisms. Based on this, it is clear that BB-12^®^ is a unique strain which can be distinguished from all other strains on the market, including strains which are so closely related that they have identical DNA fingerprints.

Possession of the complete genome sequence facilitates a number of other technologies for characterizing a strain. This includes gene expression studies and comparative genome hybridization using microarrays as well as information required to identify the specific proteins produced by a cell. This information can be used to improve production processes, identify specific compounds which support growth and provide information critical to understanding the mode of action of the probiotic properties of BB-12^®^ [3,5].

## 3. Strain Characteristics & Mechanisms

### 3.1. Acid and Bile Tolerance

Gastric acid and bile play an important role in the body’s defense against ingested microorganisms, capable of killing and controlling gastrointestinal exposure to many pathogens. However, this same defense mechanism can also disable potentially beneficial microbes. For probiotic effects that are dependent on viability and physiological activity in the intestine, the survival of the probiotic in the presence of gastric acid and bile of the upper gastrointestinal tract is critical.

Several studies have investigated the gastric acid and bile tolerance of BB-12^®^. An *in vitro* study assessed five strains of bifidobacteria for acid and bile tolerance, as well as growth on various carbohydrates. Tolerance to pH 2, pH 3, and pH 4 as well as 1% ox-gall was tested. BB-12^®^ exhibited a very good survival at all pH values, and had the best survival compared to the other strains. BB-12^®^ did not grow well at 1% bile but demonstrated high survival rates [7].

The acid tolerance of 17 strains was compared in an *in vitro* study exposing them to pH 2–5. BB-12^®^ demonstrated high survival rates. This characteristic was shown to be due in part to the low pH induction of H^+^-ATPase activity, an enzyme complex involved in maintaining intracellular pH homeostasis in bacteria [8].

In another study, 24 strains of lactic acid starter bacteria and 24 strains of probiotic bacteria were tested for tolerance to gastric juice and bile salts. BB-12^®^ showed high pH tolerance after three hours exposure at pH 3 and pH 2. The bile resistance of BB-12^®^ was moderate showing 24% growth at 1% bile compared to a control. As for deconjugation and growth in the presence of bile salts, BB-12^®^ showed both growth and deconjugation of sodium tauro-deoxycholate and sodium glyco-deoxycholate, whereas BB-12^®^ grew in the presence of sodium tauro-cholate and sodium glyco-cholate without showing any deconjugation [9].

In an internal Chr. Hansen study, a comparative evaluation of 60 human intestinal bifidobacteria isolates for gastric acid and bile survival, BB-12^®^ was demonstrated to survive both conditions just as well or better than the other tested strains (data not shown). In addition, using an artificial gut model system (TIM-1) simulating passage through gastric acid and upper intestinal bile, 60%–80% of the BB-12^®^ in a normal capsular dose remains viable (data not shown).

In conclusion, BB-12^®^ shows high gastric acid and bile tolerance compared to other bifidobacteria. The above data suggests that the majority of BB-12^®^ bacteria may survive gastric acid and bile after consumption by humans. These properties enhance the potential of BB-12^®^ to provide a health benefit to the host.

### 3.2. Bile Salt Hydrolase

**Figure 2:**
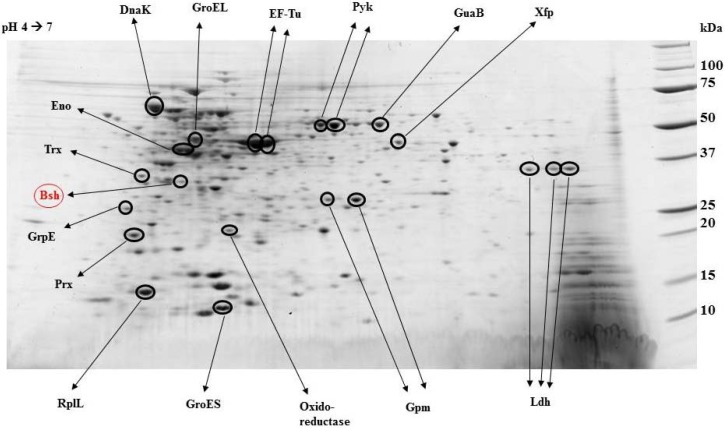
Protein studies using 2-D gel electrophoresis have documented the presence and activity of bile salt hydrolase in BB-12^®^. Marked as **Bsh** in the picture. Reproduced from Garrigues *et al*. 2005 [3] with permission of Dairy Industry Association of Australia, Copyright 2005.

The passage through the gastrointestinal tract includes different challenges for live probiotics. Following the harsh and acidic gastric environment, bile salts of the small intestine present the next challenge. BB-12^®^ contains the gene coding for bile salt hydrolase, an enzyme which is important for coping with the high bile salt concentrations in the small intestine. This enzyme is present and active in BB-12^®^ at all times, a fact which is documented by both microarray analyses and protein studies using 2-D gel electrophoresis [3] (Figure 2). Having such an enzyme ready for action will provide an advantage for the cell as it allows a quick response to high bile salt concentrations and thus facilitates the viable passage from the small intestine to the large intestine. These data suggest that BB-12^®^ is well-equipped to endure this critical passage in the gastrointestinal tract.

### 3.3. Mucus Adhesion

Adhesion of probiotic microorganisms to the intestinal mucosa is considered important for many of the observed probiotic health effects. Adhesion is considered a prerequisite for colonization, pathogen inhibition, immune interactions, and barrier function enhancement. Therefore, adhesion is one of the main selection criteria for probiotic microorganisms.

The adhesion of probiotics, commensal, and potentially pathogenic bacteria was determined *in vitro*. The adhesion models used were polycarbonate-well plates, with or without mucin, and different configurations of Caco-2 and/or HT29-MTX cell cultures. Adhesion of probiotic strains to untreated wells, as well as mucin-treated wells was high, with BB-12^®^ displaying the highest level of adhesion in both cases. Though at a lower level, BB-12^®^ also adhered to Caco-2 cultures, HT29-MTX cells, and co-cultures of Caco-2:HT29-MTX [10].

In another *in vitro* study, the adhesion properties of BB-12^®^ and other strains to fecal mucus isolated from various species including humans was tested. BB-12^®^ adhered well to all hosts tested ranging from 10% in dog mucus to 30% in human mucus [11].

Twenty-four strains of bifidobacteria were tested for their ability to bind immobilized human and bovine intestinal mucus glycoproteins. BB-12^®^ and one other *Bifidobacterium* strain had the highest level of adhesion among the tested strains. The adhesion level of BB-12^®^ to human mucus was 7.1% [12].

The adhesion of five probiotics and their combinations in the intestinal mucus of infants during and after rotavirus diarrhea and in healthy children was determined *in vitro*. Mucus was prepared from fecal samples from 20 infants during and after rotavirus diarrhea and from ten healthy age-matched children. BB-12^®^ demonstrated excellent adhesion properties with 31% in healthy children and 26.1% in infants after infection. The distinctive pattern of probiotic adherence was not influenced by rotavirus diarrhea [13].

Testing of the adherence properties of BB-12^®^ has also been evaluated at the laboratories of Chr. Hansen. In a comparative *in vitro* test of 60 human intestinal bifidobacteria isolates, BB-12^®^ was demonstrated to adhere to mucus just as well as or better than the other tested strains (data not shown).

In conclusion, BB-12^®^ has demonstrated high adherence properties in various *in vitro* settings. This evidence supports that BB-12^®^ possesses the capability to transiently colonize the mucosal surfaces in the intestine, persist at these sites, and thereby increase the possibility of delivering beneficial health effects.

### 3.4. Pathogen Inhibition

Pathogens are microorganisms that may cause disease in their host. The ability to inhibit pathogens is one of the three main mechanisms of probiotics, barrier function enhancement and immune interactions being the other two. It is proposed that pathogen inhibition is facilitated through multiple mechanisms including: production of inhibitory substances (organic acids, H_2_O_2_, bacteriocins); nutrient competition; toxin removal/degradation; competition for sites of adherence (mucus, cell receptors); co-aggregation and virulence modulation; and induction of host immune responses.

An *in vitro* study compared four different microorganisms including BB-12^®^ with regards to production of antagonistic substances, amongst others. *Bacillus cereus*, *Clostridium difficile*, *Clostridium perfringens* Type A, *Escherichia coli* ATCC 4328, *Enterococcus faecalis*, *Listeria monocytogenes*, *Pseudomonas aeruginosa*, *Salmonella enterica* subsp *enterica* serovar Typhimurium, *S. enterica* subsp. *enterica* serovar Typhi, *Shigella flexneri*, *Shigella sonnei*, and *Candida albicans* were used in the antagonism assay. Only BB-12^®^ and one other bacteria strain produced inhibitory zones against the pathogens. BB-12^®^ displayed antagonistic activity against eight out of the twelve tested pathogens and the inhibitory zones produced by BB-12^®^ were in general larger with the only exception of *S. flexneri* [14].

Batch and continuous culture anaerobic fermentation systems, inoculated with human feces, were utilized to investigate the antimicrobial actions of two probiotics combined with prebiotics. BB-12^®^ combined with a mixture of oligofructose and xylo-oligosaccharides was tested against *E. coli* and *Campylobacter jejuni*. In batch fermenters, both *E. coli* and *C. jejuni* were inhibited by BB-12^®^ combined with prebiotics. In continuous culture BB-12^®^ and prebiotics inhibited *C. jejuni*. The results suggested that acetate and lactate produced by BB-12^®^ directly were conferring antagonistic action, rather than as a result of pH lowering.

The ability of commercial probiotic strains currently marketed in European countries, to inhibit, compete with and displace the adhesion of selected potential pathogens to immobilized human mucus was investigated in an *in vitro* study. Bacterial pathogens were; *Bacteroides vulgatus*, *Clostridium histolyticum*, *C. difficile*, *E. coli* K2, *Enterobacter aerogenes*, *L. monocytogenes*, *S. enterica* serovar Typhimurium, and *Staphylococcus aureus*. BB-12^®^ was able to adhere to the human mucus and inhibited all pathogens but *E. coli*. BB-12^®^ demonstrated good displacement of *C. difficile*, *B. vulgatus*, *E. aerogenes*, *L. monocytogenes* and to a minor degree *C. histolyticum*, *S. enterica* and *S. aureus* [15].

In addition, a competition and exclusion experiment for mucus adherence also demonstrated the ability of BB-12^®^ to reduce binding of pathogens. An *in vitro* study set out to investigate the protective effect of BB-12^®^ and *L. rhamnosus* LGG^®^, alone and in combination, on the adhesion of pathogenic strains to pig intestinal mucus. Pathogens used were *S. enterica* serovar Typhimurium, *C. perfringens*, *C. difficile*, and *E. coli* K2. BB-12^®^ and LGG^®^ in combination enhanced the adhesion of each other, mainly in large intestinal mucus. Treatment of intestinal mucus with BB-12^®^ and LGG^®^, alone or in combination significantly reduced adhesion of the tested pathogens [16].

In conclusion, these studies show that BB-12^®^ is capable of inhibiting important gastrointestinal pathogens through production of antimicrobial substance as well as through competition for mucosal adhesion.

### 3.5. Barrier Function Enhancement

Barrier function enhancement is one of the central and generally accepted mechanisms of probiotics. Maintenance of an intact and functional mucus layer and epithelial cell lining in the gastrointestinal tract is critical in order to stay fit and healthy.

An *in vitro* study aimed at testing whether or not fermentation products from probiotics and prebiotics affected tight junction integrity in a Caco-2 cell line model; this was done by measuring the transepithelial electric resistance (TER, Ω/cm^2^) of the Caco-2 cells. Fermentation products from BB-12^®^ increased tight junction strength significantly above that of the untreated control, and in all cases, fermentation products from BB-12^®^ induced the greatest increase in TER compared to other strains tested. These *in vitro* changes indicate that BB-12^®^ may increase tight junction strength and protect against disruption of the epithelial barrier function [17].

### 3.6. Immune Interactions

Immune interaction is increasingly being acknowledged as a substantial probiotic mechanism. Probiotics are capable of communicating with and affect the immune system through immune cells located in the intestine. Seventy to eighty percent of the immune cells are associated with the gut mucosa.

Several studies have demonstrated the immune modulating effect of BB-12^®^. The effect of twelve *Bifidobacterium* strains on the maturation process of dendritic cells derived from human monocytes was studied *in vitro*. Furthermore, proliferation of peripheral blood mononuclear cells and cytokine expression were evaluated. Maturation due to lipopolysaccharide treatment was used as reference. BB-12^®^ was able to induce maturation of dendritic cells to a similar or even higher degree than LPS measured by surface expression markers. Cell-free supernatant only had a weak effect or no effect on maturation of dendritic cells. Expression of cytokines varied to a great extent depending on the strain, however, BB-12^®^ demonstrated induction of IL-12 and TNF-α to a high degree and IL-10 to a low degree. In PBMCs, BB-12^®^ induced high levels of IL-10, IFN-γ and TNF-α [18].

The ability of nine different probiotic strains to induce maturation and cytokine/chemokine expression in human dendritic cells at various concentrations was studied. BB-12^®^ was able to induce all cytokines tested (IL-1β, IL-6, IL-10, IL-12 and IFN-γ). The response was dose-dependent and increased with higher dose. With regards to chemokines, BB-12^®^ induced CCL20 in a dose-dependent manner [19].

An *in vitro* study investigated if fecal precipitates, obtained during consumption of BB-12^®^, induce an anti-inflammatory response in a murine macrophage-like cell line. Fecal precipitates tended to elicit a higher TNF-α response during the period of BB-12^®^ consumption, compared to pre- and post-consumption. No change in response was observed for IL-1α and IL-10 [20].

In conclusion, these data show that BB-12^®^ is able to interact with the immune cells and demonstrate that BB-12^®^ may have a beneficial impact on the immune function.

## 4. Efficacy

### 4.1. Proven Efficacy

BB-12^®^ is the world’s most documented probiotic *Bifidobacterium*. It is described in more than 300 scientific publications out of which more than 130 are publications of clinical studies. Dating back to 1987, BB-12^®^ has been tested in clinical trials for more than 25 years [21]. BB-12^®^ has been tested in clinical trials including subjects from preterm infants to elderly, and it has been administered in dosages up to 100 billion CFU/day.

It is a prerequisite for a probiotic to have documented its beneficial effect on the host in clinical studies. BB-12^®^ has proven its beneficial health effect both within gastrointestinal health and immune function in numerous clinical studies.

### 4.2. Survival in the Gastrointestinal Tract

Some probiotic mechanisms presuppose viability and physiological activity of the probiotic at the target site. Since the target site may not be well-defined, and due to difficulties measuring the viability *in situ*, fecal recovery is often used to confirm viability of probiotics in the gastrointestinal tract.

A placebo-controlled, cross-over study evaluated the effect of a synbiotic yogurt containing BB-12^®^ and inulin on recovery of BB-12^®^. Fecal samples were collected from 46 volunteers and recovery, as well as changes in the microbiota were monitored using real-time polymerase chain reaction (PCR). BB-12^®^ was recovered in the fecal samples and could be detected in feces up to two weeks after intake. A live/dead PCR procedure indicated that >90% of the detected BB-12^®^ in the fecal samples was alive [22].

In a randomized, placebo-controlled, double-blinded, parallel dose-response study 71 healthy young adults were assigned into five groups. The subjects received either placebo or a mixture of the two probiotics in the four concentrations of 10^8^–10^11^ CFU/day for three weeks. The fecal recovery of BB-12^®^ increased significantly with increasing dose. In the high dosage group, BB-12^®^ was recovered in 13 out of 15 volunteers [23].

In a study including 14 volunteers the intestinal survival and persistence of BB-12^®^, F19^®^ and *Lactobacillus acidophilus* NCFB 1748 consumed in fermented milk was tested. Fecal recovery was detected by randomly amplified polymorphic DNA (RAPD) fingerprinting analysis of isolates from lactobacilli-selective media. BB-12^®^ and F19^®^ survived well through the gastrointestinal tract and were detected in 79% and 100%, respectively, of the study subjects after ingestion [24].

Another study included 30 healthy adults who were divided into three intervention groups: galacto-oligosaccharide (GOS), or GOS and BB-12^®^, or BB-12^®^ alone. Fecal samples were collected before the intervention, and after the intervention. BB-12^®^ was recovered from feces using RAPD fingerprinting. Isolates having an identical RAPD fingerprint with BB-12^®^ were detected in high numbers in both the BB-12^®^ group and the GOS + BB-12^®^ group indicating good survival of BB-12^®^ through the gastrointestinal tract. BB-12^®^ was found in 40% of the volunteers from the BB-12^®^ group and the GOS + BB-12^®^ group one week after the intervention and in 20% two weeks after the intervention [25].

In conclusion, these data show that not only does BB-12^®^ survive very well during the passage through the gastrointestinal tract, it also transiently colonizes the colon. The data suggest a dose related recovery of BB-12^®^.

### 4.3. Modulation of Intestinal Microbiota

The human large intestine is host to a wide variety of bacteria, with bifidobacteria being prominent members of this complex ecosystem. Bifidobacteria and lactobacilli are generally believed to contribute to gastrointestinal health. During old age, bifidobacteria and lactobacilli begin to decline in number, coinciding with proliferation of other bacterial groups, including clostridia and members of the *Enterobacteriaceae* family, which are believed to have adverse effects on gastrointestinal health. Probiotics capable of controlling proliferation of undesirable bacteria and increasing the levels of bifidobacteria and lactobacilli in the colon are considered beneficial.

Several clinical studies have shown that BB-12^®^, alone or in combination with other probiotics or ingredients, is associated with an increase in beneficial bacteria and a reduction in potentially pathogenic bacteria.

A double-blind, randomized, placebo-controlled dose-response study investigated the impact of a four-week consumption of BB-12^®^, 8 × 10^9^ or 38 × 10^9^ CFU/day, *L. acidophilus* LA-5^®^, 1 × 10^9^ CFU/day, and green tea extract in fermented milk on fecal bacterial counts in 58 healthy adults [26]. Quantitative PCR results showed significant increases in bifidobacteria counts in the active groups compared to baseline. Numbers of viable fecal lactobacilli were significantly higher and those of enterococci were significantly lower after the intervention when compared to placebo.

In an open-label, single arm study, the effect of two weeks intake of BB-12^®^ (total of 1 × 10^11^ CFU/day), *L. paracasei* subsp. *paracasei* F19^®^, and *L. acidophilus* NCFB 1748 in fermented milk on the intestinal microbiota of 15 healthy adults was studied [24]. The probiotic strains tended to transiently increase the total numbers from baseline of bifidobacteria and lactobacilli in feces. No change in clostridia, enterobacteriacea, or enterococci was detected.

A three arm, single blinded study randomized 30 healthy adults to either 3 × 10^10^ CFU/day of BB-12^®^, BB-12^®^ in combination with 4 g GOS, or 4 g GOS alone for two weeks [25]. Outcome measures were selected components of the fecal microbiota by culture methods. During the intervention period, the mean numbers of bifidobacteria increased slightly in all study groups. The increase was higher in the BB-12^®^ and GOS group than in the BB-12^®^ group. The increases were statistically significant. No changes in lactic acid bacteria, *C. perfringens*, or coliforms were detected during the study.

In conclusion, these studies show that consumption of BB-12^®^ facilitates an increase of the total number of bifidobacteria and may inhibit some undesirable bacteria in the gastrointestinal microbiota. This is believed to support a healthy microbiota in the gut.

### 4.4. Gastrointestinal Function

#### 4.4.1. Bowel Function

Regular bowel movements, natural transit time and normal stool consistency are part of a well-functioning bowel. However, the boundaries for normal bowel function are wide and vary to a large extent from person to person. The normal range for bowel movements is five to 14 times a week, with outer boundaries of three to 21 times a week. A frequency higher or lower is considered diarrhea or constipation, respectively. The passage time for food through the gastrointestinal tract is normally within half a day to three days. Lazy tummy or constipation is a widely experienced challenge, especially in the elderly population. Probiotics may support the bowel function in a beneficial way by increasing bowel movement or transit time, or by softening of the stools.

**Figure 3:**
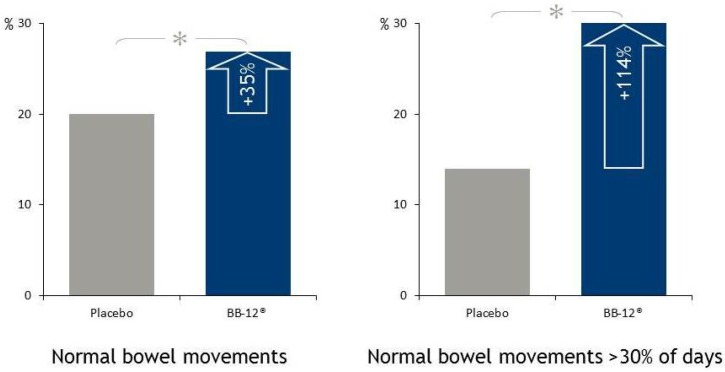
In a randomized, double-blind, placebo-controlled study by Pitkala *et al*. 2007 [27], BB-12^®^ was shown to improve bowel movement by 35%. The number of subjects experiencing normal bowel movements in more than 30% of the days was increased by 114% in the BB-12^®^ group. Reproduced from Pitkala *et al*. 2007 [27] with permission of the author, Copyright 2007.

The effect of a fermented oat drink with 1 × 10^9^ CFU/day of BB-12^®^, or two *B. longum* strains, or placebo on bowel movement was tested in 209 elderly nursing home residents in a double-blind, randomized, placebo-controlled study [27]. Residents received the intervention for one to seven months depending on the length of the stay in the nursing home. The group receiving BB-12^®^ had significantly more days with normal bowel movements relative to total number of days with bowel movements: 26.9% for BB-12^®^ and 20% for placebo. The number of subjects experiencing normal bowel movements in more than 30% of the days was increased by 114% in the BB-12^®^ group (Figure 3).

Studies in healthy adults have demonstrated that BB-12^®^ increases stool frequency and softens the consistency of the stool. In a double-blind, randomized, placebo-controlled, cross-over design, BB-12^®^ in fermented milk was given to healthy females in a dosage of 1 × 10^9^ CFU/day for two weeks [28]. In this study, 41 females had an average stool frequency in the BB-12^®^ period of 8.8 per two weeks compared to 8.0 in the placebo period. When the subjects were divided into constipation (less than eight times per two weeks) and no constipation tendency groups, stool frequency was significantly higher in the BB-12^®^ period compared to the placebo period in the constipation tendency group.

In a similar design, BB-12^®^ in fermented milk at a dosage of 4 × 10^9^ CFU/day was given for two weeks to 35 healthy females [29]. A non-significant increase in stool frequency was found between the BB-12^®^ period and the placebo in all subjects, whereas a statistically significant increase was found between the BB-12^®^ period and the placebo period in constipated subjects with stool frequency of four times per week or less.

In a single-arm placebo-controlled study, 30 healthy adults were given BB-12^®^ in fermented milk at a dosage of 5.2 × 10^9^ CFU/day for two weeks, followed by a two week period of placebo with a wash-out period in between [30]. One month after the placebo period, BB-12^®^ in fermented milk at a higher dosage (15 × 10^9^ CFU/day) was given for two weeks. The inclusion criteria were a stool frequency of less than four times weekly. The primary outcome measure was stool frequency. Stool frequency was higher in both BB-12^®^ low-dosage and high-dosage periods compared to the placebo period.

In conclusion, the above studies demonstrate that BB-12^®^ improves bowel function, particularly in subgroups with mild constipation.

#### 4.4.2. Diarrhea in Infants and Children

Diarrhea is a serious cause of infant morbidity and mortality, and the development of preventive measures remains an important goal. Bifidobacteria as well as other lactic acid producing bacteria have been shown to have a protective effect against both acute and persistent diarrhea.

A multicenter, double-blind, placebo controlled study evaluated the efficacy of a milk formula supplemented with BB-12^®^ in the prevention of acute diarrhea in 90 healthy infants younger than eight months living in residential nurseries or foster care centers. There was a tendency toward a decrease in the incidence of diarrhea, with 28.3% of the infants receiving BB-12^®^ experiencing acute diarrhea compared to 38.6% in the placebo group. Number of days with diarrhea was statistically lower in the BB-12^®^ group as well as a lower day-probability of diarrhea. These results suggest that BB-12^®^ have a protective effect against diarrhea [31].

In a double-blind, placebo-controlled study, young children who were admitted to a chronic medical care hospital were randomized to receive a standard infant formula or the same formula supplemented with BB-12^®^ and *Streptococcus thermophilus* TH-4^®^ [32]. Children were evaluated daily for occurrence of diarrhea, and fecal samples were analyzed for rotavirus antigen by enzyme immunoassay. Fecal samples were also obtained during an episode of diarrhea for virological and bacteriological analysis. Fifty-five infants were evaluated for a total of 4447 patient-days during 17 months. Eight (31%) of the 26 children who received the control formula, and two (7%) of the 29 who received the supplemented formula, developed diarrhea during the course of the study. Ten (39%) of the subjects who received the control formula and three (10%) of those who received the supplemented formula shed rotavirus at some time during the study. These results suggest that supplementation of infant formula with BB-12^®^ and *S. thermophilus* TH-4^®^ can reduce the incidence of acute diarrhea and rotavirus shedding in children admitted to hospital.

In conclusion, these studies demonstrate that BB-12^®^ may have a beneficial effect on both the incidence and duration of diarrhea in infants and children.

#### 4.4.3. Antibiotic-Associated Diarrhea

Treatment with antibiotics may cause serious side effects. Typically the disturbance of the gastrointestinal microbiota caused by the antibiotics leads to vomiting and diarrhea. Probiotics have shown to be able to reduce the side effects and in addition increase the completion rate of the antibiotic treatment. Furthermore, probiotics may accelerate recovery after the antibiotic treatment.

A randomized, double-blind, placebo-controlled study evaluated the efficacy of BB-12^®^ and LA-5^®^ in the prevention of antibiotic-associated diarrhea (AAD) in 343 patients during a seven day antibiotic treatment. Fourteen days of intervention was evaluated by symptom diary card for AAD assessment. After 14 days treatment, incidence of AAD in the probiotic group was significantly reduced to 10.8% compared to 15.56% in the placebo group. The duration of diarrhea was significantly less (2.32 days) in the probiotic group compared to placebo group (4.58 days). Incidence of severe diarrhea was significantly higher in the placebo group (96%) than the probiotic group (31.6%). These results show that BB-12^®^ and LA-5^®^ can effectively reduce the duration and severity of AAD [33].

To investigate matrix-specificity of probiotic effects and particularly the improvement of AAD, a controlled, randomized, double-blind study was performed. Eighty-eight *Helicobacter pylori*-infected but otherwise healthy subjects were given fermented milk containing BB-12^®^ and LA-5^®^, pasteurized fermented milk with BB-12^®^ and LA-5^®^, or acidified milk (control) for eight weeks. During week five, a *H. pylori* eradication therapy was performed. *H. pylori* activity was measured via urea breath tests and AAD and other gastrointestinal complaints were recorded by validated questionnaires. Subjects that were given fermented milk with live BB-12^®^ and LA-5^®^ had significantly decreased duration of AAD (four days) compared to subjects given pasteurized fermented milk (ten days) or control (ten days). Moreover, probiotics significantly improved gastrointestinal symptoms [34].

One hundred and sixty *H. pylori* infected patients were randomized into a triple-plus-probiotic-group or a triple-only-group, receiving one week of triple therapy with or without five weeks of supplementation with fermented milk containing BB-12^®^ and LA-5^®^. Bifidobacteria was tested in stool samples. A significantly higher proportion in the probiotic group completed the seven-day treatment than in the control group (68% *vs*. 44%). Common side effects such as vomiting, constipation, diarrhea and metallic taste were significantly decreased in the probiotic group. *H. pylori* eradication rates were significantly higher in the probiotic group. After triple therapy both groups observed depletion of bifidobacteria in stools. However probiotics restored the level of bifidobacteria in four weeks, as opposed to the control group. This shows that BB-12^®^ and LA-5^®^ can reduce the side effects of antibiotic treatment, aid treatment compliance, improve the eradication rates of *H. pylori* and restore the microbiota [35].

The antagonistic effect of BB-12^®^ and LA-5^®^ against *H. pylori* has also been confirmed in other studies [36,37].

In conclusion, these studies demonstrate that BB-12^®^ in combination with LA-5^®^ can reduce the incidence and duration of antibiotic-associated diarrhea significantly. In addition BB-12^®^ and LA-5^®^ may suppress *H. pylori* and support restoration of the microbiota in *H. pylori* positive subjects.

### 4.5. Immune Function

#### 4.5.1. Respiratory Infections

Studying the immune system in healthy humans poses a special challenge. The immune system carries a high degree of buffering capacity for several components, which makes it difficult to interpret or predict the exact response at a given time [38]. The use of a model infection is therefore considered to provide the best method for exploring the function and the response of the immune system in healthy humans [38,39]. One of the suggested methods is the use of a vaccine containing killed or attenuated pathogens that will result in a specific immune response. Response to such a challenge can be used as an indicator of an integrated immune response.

Probiotics may interact with the immune system in various ways, e.g., by increasing local and systemic antibody production, by increasing immune cell activity, by modulating signals in epithelial and immune cells, and by induction of phenotypic changes in dendritic cells.

**Figure 4:**
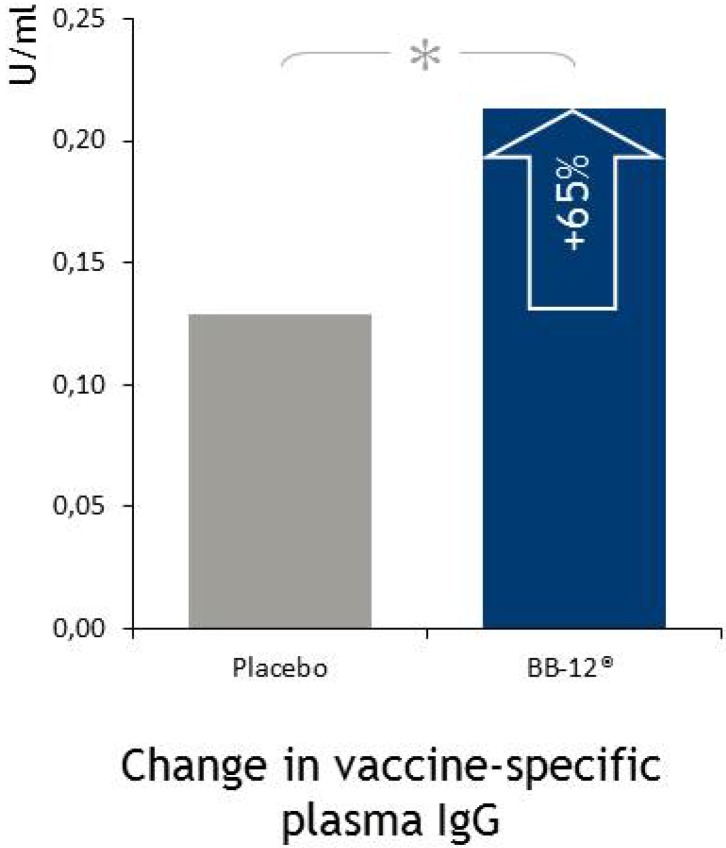
In a randomized, double-blind, placebo-controlled study by Rizzardini *et al*. 2012 [40], BB-12^®^ was shown to improve immune function. Reproduced from Rizzardini *et al*. 2012 [40] with permission of Cambridge University Press, Copyright 2012.

A randomized, placebo-controlled, double-blind human study investigated the impact of BB-12^®^ on the functional capacity of the immune system in healthy humans using a vaccination model. In this study, 106 subjects were given BB-12^®^ or placebo for six weeks. After two weeks subjects received an influenza vaccination. Plasma and saliva samples were collected at baseline and after six weeks for analysis of influenza specific and total antibodies, cytokines IL-2, IL-10 and INF-γ, and innate immune parameters. BB-12^®^ increased the influenza specific antibody responses compared to placebo (Figure 4) and the number of subjects obtaining a minimum two-fold increase in antibody levels was significantly greater in the probiotic group. No differences were found for cytokines or innate immune parameters. Adverse event incidence and pattern was similar between groups, and tetanus-specific IgG did not change after the intervention indicating that supplementation with BB-12^®^ only elicits specific immune responses [40].

A study investigated the effect of BB-12^®^ and LGG^®^ on health-related quality of life during upper respiratory infections. The study assessed how probiotics affect duration of common cold, severity, and the impact of symptoms on daily life. One hundred ninety eight college students were randomized to receive either placebo or BB-12^®^ and LGG^®^ for twelve weeks. Each day, students completed a survey to assess the effect of the probiotic supplementation. The median duration of upper respiratory infections was significantly shorter by two days, and median severity score was significantly lower by 34% in the probiotic group compared to placebo. Number of missed work-days was not different between groups. However, the probiotics group missed 0.2 fewer school days compared to the placebo group. The study shows that BB-12^®^ and LGG^®^ shortens the duration of colds and minimizes the severity for college students, and reduces missed school days [41].

**Figure 5:**
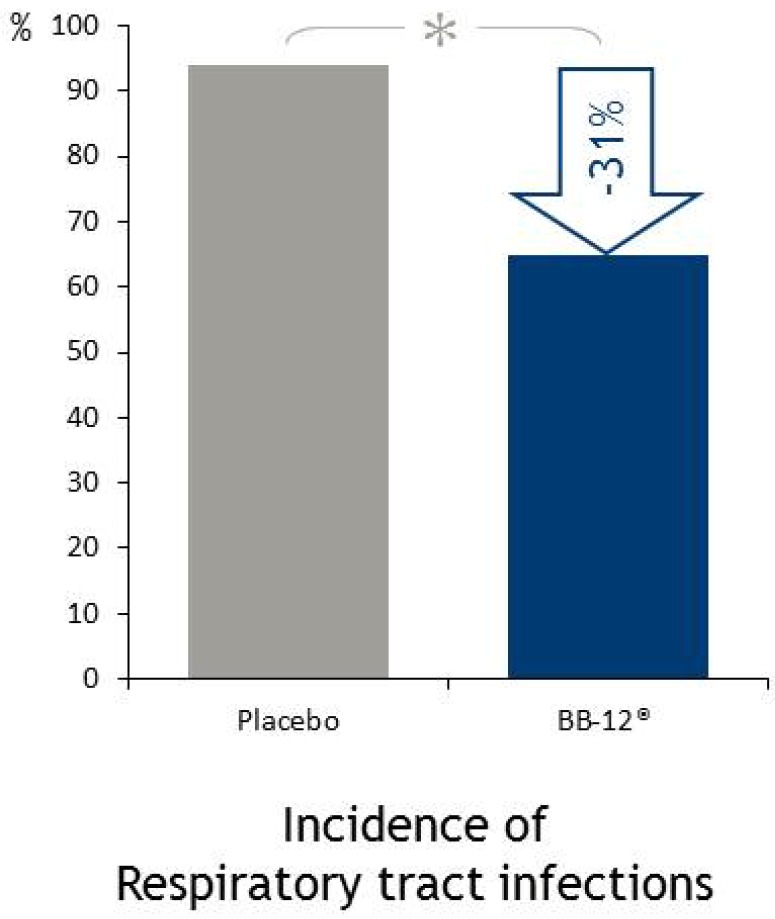
BB-12^®^ reduced the risk of respiratory tract infections in a study by Taipale *et al*. 2011 [42]. In this randomized, double-blind, placebo-controlled BB-12^®^ was administered using a slow-release tablet in a pacifier. Reproduced from Taipale *et al*. 2011 [42] with permission of Cambridge University Press, Copyright 2011.

In a randomized, placebo-controlled and double-blind study, the effect of BB-12^®^ on the risk of acute infections in infants was investigated. BB-12^®^ or placebo was administered to 109 infants less than two months of age until the age of eight months. Signs and symptoms of acute infections were registered. In this study there was no effect on gastrointestinal infections or otitis media, however, fewer respiratory infections were reported in the BB-12^®^ group compared to placebo (Figure 5) [42].

Another clinical study also showed reduction of acute infections in infancy. In this study 81 formula fed infants aged less than two months were randomized to either probiotics (BB-12^®^ and LGG^®^) or placebo for twelve months in a double-blind design. Incidence of early infections and antibiotic use at seven months of age was lower in the group given probiotics compared to placebo group. During the first year of life, infants receiving probiotics also had fewer recurrent respiratory infections [43].

In conclusion, these data demonstrate that supplementation with BB-12^®^ may increase the body’s resistance to common infections by strengthening the specific immune response to an immune challenge and that BB-12^®^ can reduce incidence and duration of respiratory infections.

## 5. Conclusions

*In vitro* and animal studies have contributed to the understanding of strain characteristics and mechanisms of BB-12^®^. Characteristics include excellent acid and bile tolerance, bile salt hydrolase, and strong adherence properties. Pathogen inhibition, barrier function enhancement, and immune interactions are mechanisms that have been demonstrated for BB-12^®^.

Beneficial health effects of BB-12^®^ have been demonstrated through clinical research. Survival of BB-12^®^ through the gastrointestinal tract has been demonstrated and BB-12^®^ has been shown to support a healthy gastrointestinal microbiota. Furthermore, BB-12^®^ has been shown to improve bowel function, to have a protective effect against diarrhea, and to reduce side effects of antibiotic treatment. In terms of immune function, clinical studies have shown that BB-12^®^ increases the body’s resistance to common respiratory infections as well as reduces the incidence of acute respiratory tract infections.

In conclusion BB-12^®^ has well-established probiotic characteristics and proven beneficial health effects within gastrointestinal health and immune function.
